# Stromal Cell-Derived Factor 1 Gene Polymorphism Is Associated with Susceptibility to Adverse Long-Term Allograft Outcomes in Non-Diabetic Kidney Transplant Recipients

**DOI:** 10.3390/ijms150712495

**Published:** 2014-07-15

**Authors:** Chung-Jieh Wang, Jen-Pi Tsai, Shun-Fa Yang, Jong-Da Lian, Horng-Rong Chang

**Affiliations:** 1 Institute of Medicine, Chung Shan Medical University, Taichung 402, Taiwan; E-Mails: chung-jieh@yahoo.com.tw (C.-J.W.); abi0918@yahoo.com.tw (J.-P.T.); ysf@csmu.edu.tw (S.-F.Y.); 2 Department of Nephrology, Buddhist Dalin Tzu Chi General Hospital, Chiayi 622, Taiwan; 3 Division of Nephrology, Department of Internal Medicine, Chung Shan Medical University Hospital, Taichung 402, Taiwan; E-Mail: cshy525@csh.org.tw

**Keywords:** gene polymorphism, stromal cell-derived factor 1, renal transplantation

## Abstract

Although the genetic polymorphism of Stromal Cell-Derived Factor 1 (SDF-1) is associated with higher mortality of liver allograft recipients, the role of SDF-1 in the modulation of renal allograft outcomes is unclear. Between March 2000 and January 2008, we recruited 252 non-diabetic renal transplant recipients (RTRs). Baseline characteristics and blood chemistry were recorded. Genomic DNA extraction with polymerase chain reaction-restriction fragment length polymorphism was utilized to analyze the genetic polymorphisms of SDF-1 (rs1801157). The influence of SDF-1 on an adverse renal allograft outcome, defined as either a doubling of serum creatinine, graft failure, or patient death was evaluated. Sixteen patients with the SDF-1 AA/AG genotype and nine with the SDF-1 GG genotype reached an adverse outcome. According to Kaplan-Meier analysis, patients carrying the SDF-1 AA/AG genotype or A allele showed a significantly higher risk of reaching an adverse outcome than those carrying the SDF-1 GG genotype or G allele (*p* = 0.041; *p* = 0.0051, respectively; log rank test). Stepwise multivariate Cox proportional regression analysis revealed that patients carrying the SDF-1 AA/AG genotype and A allele had a 2.742-fold (95% CI. 1.106–6.799, *p* = 0.03) and 2.306-fold (95% CI. 1.254–4.24, *p* = 0.008) risk of experiencing an adverse outcome. The SDF-1 AA/AG genotype and A allele have a detrimental impact on the long-term outcome of RTRs.

## 1. Introduction

The outcome of renal allograft is determined by a combination of risk factors including immunologic risk factors, such as delayed graft function (DGF), acute rejection, hepatitis B (HBV), and hepatitis C (HCV) infection as well as non-immunologic risk factors, such as hypertension (HTN), hyperlipidemia, and hyperglycemia [1,2,3]. Recently, it has been reported that single nucleotide polymorphisms (SNPs) contribute to short and long term outcomes [4,5], including DGF, chronic allograft dysfunction, and graft rejection [6,7].

Chemokines, which are involved in leukocyte migration during tissue inflammation [8], are known to be required for development and tissue homeostasis [8]. Anders *et al.* reported that SDF-1-CXCR4 function is also necessary to maintain homeostatic tissue renewal and regeneration upon renal injury [8]. SDF-1 (rs1801157) is located on chromosome 10q 11.1. A guanine to adenine (G to A) SNP at position 801 of the 3' untranslated region has been found to result in a SDF-1 chemokine gene polymorphism [9]. This SNP was first reported in a study by Winkler *et al*., which demonstrated that the onset of acquired immunodeficiency syndrome (AIDS) was delayed in 2587 patients with SDF-1 3' A/3' A (homozygous) [10]. A similar finding was observed in a study conducted by Gianesin *et al.*, who showed that children born to a human immunodeficiency virus type 1 (HIV-1) seropositive mother carrying the SDF-1 GA genotype had a higher risk of late AIDS than those carrying a GG genotype [11].

This SDF-1 A/A or AG gene polymorphism has been suggested to alter the production of SDF-1 [12]. Soriano *et al.* found that in: exposed but not infected HIV-1 patients, in HIV-1 positive patients, and all patients, there were no significant differences between wild-type homozygotes and SDF-1 3' A heterozygotes, but each one of these two groups showed very significantly increased plasma SDF-1 concentrations compared with those in SDF-1 3' A homozygotes [12]. In addition, a study from Italy conducted by Xiao *et al.* showed that SDF-1 gene variation has an influence on SDF-1 level [13]. These studies indicate that variant SDF-1 genotypes play a role in the functional expression of this gene.

In addition to these studies showing the relationship of the SDF-1 SNP with infection, this SNP appears to be associated with a higher mortality among liver allograft recipients [14]. Additionally, along with its principal receptor CXCR4, SDF-1 regulates trafficking of stem cells during development, homeostasis, inflammation, and regeneration [15]. Togel *et al.* conducted an ischemic/reperfusion induced acute kidney injury and reported that SDF-1 is a major factor involved in kidney repair through the recruitment of cells involved in tissue regeneration [16]. Moreover, an anti-SDF-1 antibody was found to retard the process of chronic allograft nephropathy [17]. However, there remains limited knowledge of the role of SDF-1 and its genetic polymorphisms in the modulation of renal allograft.

Hyperglycemia has an adverse effect on graft function and patient survival [18,19]. Indeed, we recently found that pre-diabetic patients and post-transplant diabetic mellitus (PTDM) had more adverse long-term renal allograft outcomes than those without DM [20]. In order to investigate the influences of genetic polymorphisms of SDF-1 and to eliminate the adverse effects of DM, we recruited renal transplant recipients (RTRs) who were free of DM throughout the study. We hypothesized that the variant genetic polymorphism of SDF-1 could contribute to the long-term renal allograft outcome; this information could be useful in identifying individuals at higher risk of developing adverse outcomes.

## 2. Results

**Figure 1:**
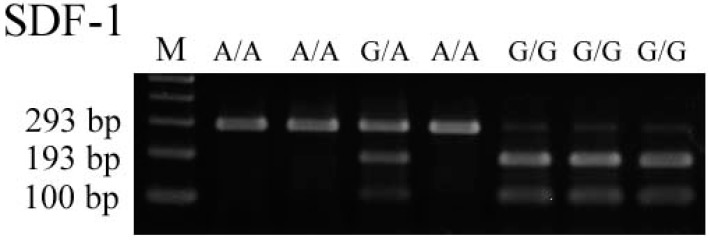
Wild type homozygous allele (G/G) yielded 100- and 193-bp products, the heterozygous alleles (G/A) yielded 100-, 193-, and 293-bp products, while the mutated type homozygous alleles (A/A) yielded a 293-bp product.

For SDF-1, the wild-type homozygous allele (G/G) yielded 100- and 193-bp products, the heterozygous alleles (G/A) yielded 100-, 193-, and 293-bp products, while the mutated type homozygous alleles (A/A) yielded a 293-bp product. These results are summarized in Figure 1. The SDF-1 genotype was in agreement with the HWE (AA:AG:GG = 19:102:131; χ^2^ = 0.019, *p* = 0.889). As illustrated in Table 1, the baseline clinical characteristics were comparable between RTRs who possessed the SDF-1 AA/AG genotype and those who possessed SDF-1 GG genotype.

**Figure 2:**
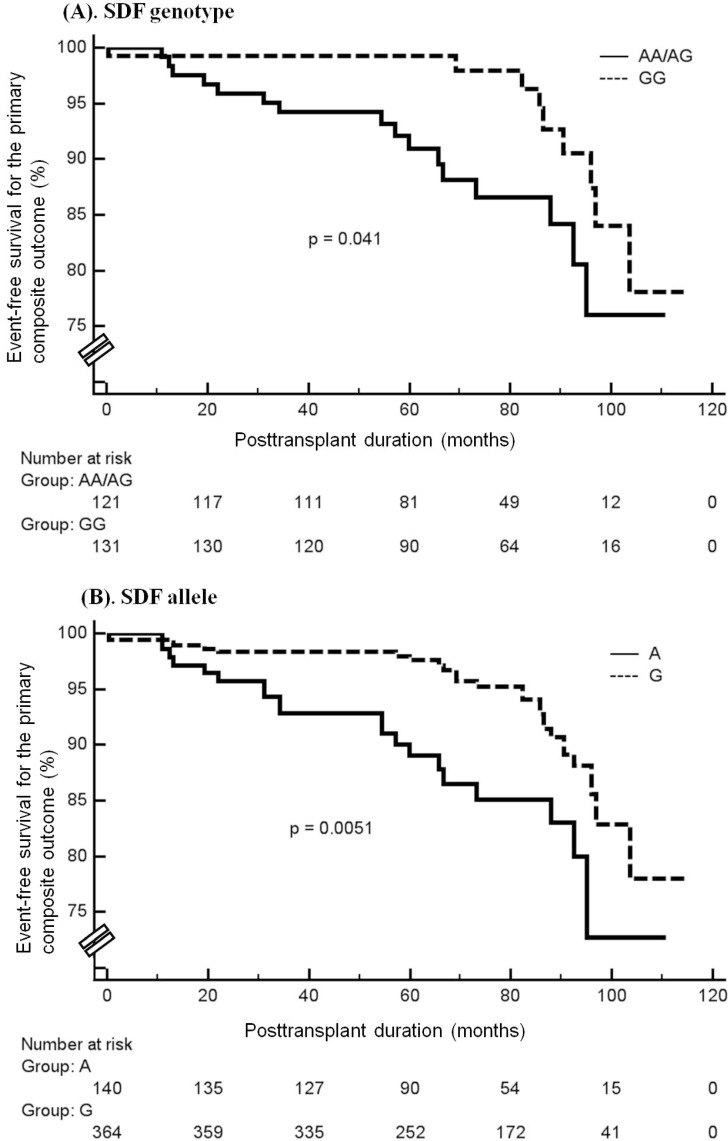
Kaplan-Meier curve and log-rank test comparing the cumulative event-free probability of time to an adverse outcome between patients with SDF-1 genotype AA/AG or GG (**A**) and those with the SDF-1 allele A or G (**B**).

A total of 25 RTRs reached the primary outcome, 16 (13.2%) of whom had the SDF-1 AA/AG genotype while the remaining 9 (6.9%) had the SDF-1 GG genotype. Compared to the RTRs with SDF-1 GG genotype, RTRs who carried the SDF-1 AA/AG genotypes showed a non-significant trend for being more likely to reach the primary outcome (AA/AG:GG = 13.2%:6.9%; *p* = 0.092). According to Kaplan-Meier analysis, RTRs who carried the SDF-1 GG genotype or G allele showed a significantly lower risk of reaching the primary composite outcome compared to those with the SDF-1 genotype AA/AG or allele A (*p* = 0.041; *p* = 0.0051, respectively; log rank test, Figure 2A,B). 

**Figure 3:**
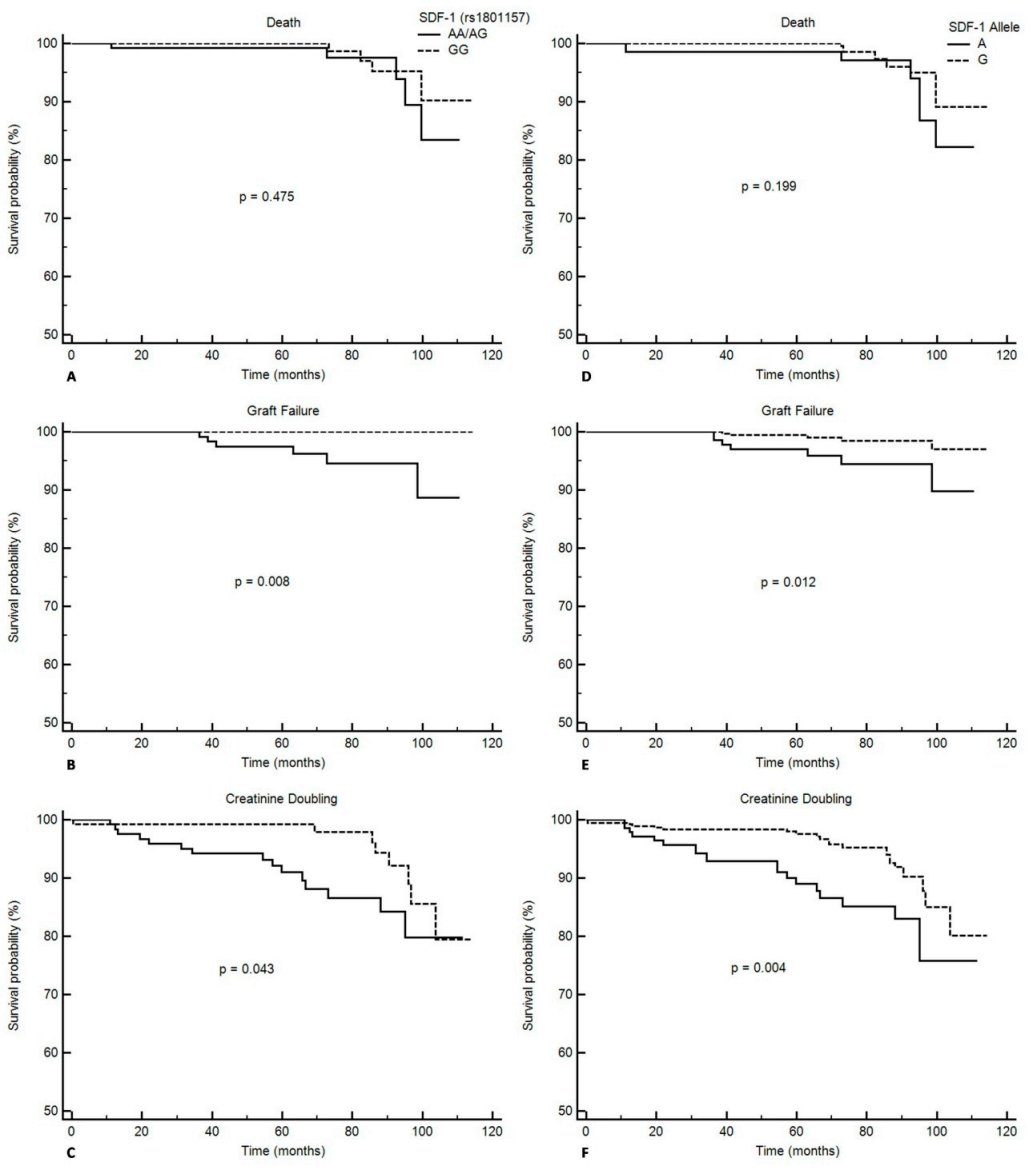
Kaplan-Meier curve and log-rank test comparing the cumulative event-free probability of time to individual adverse outcome, including death (**A**, **D**); graft failure (**B**, **E**); and creatinine doubling (**C**, **F**) between patients with SDF-1 genotype AA/AG or GG and those with the SDF-1 allele A or G.

By analyzing the Kaplan-Meier curve, RTRs that carried variant SDF-1 genotypes or allele did not show significant difference of development of death (AA/AG *vs.* GG, *p* = 0.475; A *vs.* G, *p* = 0.199) but RTRs that carried the SDF-1 AA/AG genotype or A allele had worse graft survival (AA/AG *vs.* GG, *p* = 0.008; A *vs.* G, *p* = 0.012) and a higher risk of creatinine doubling (AA/AG *vs.* GG, *p* = 0.043; A *vs.* G, *p* = 0.004) (Figure 3).

After adjustment for age, gender, HLA, CV events, HCV infection, and usage of immunosuppressive regimens, stepwise multivariate Cox proportional regression analysis revealed that RTRs carrying the SDF-1 AA/AG genotype and an A allele had a 2.742-fold (95% CI. 1.106–6.799, *p* = 0.03) and 2.306-fold (95% CI. 1.254–4.24, *p* = 0.008) risk at the development of primary outcome, respectively (Table 2).

**Table 1:** Baseline characteristics of patients with SDF-1 AA/AG or GG genotype.

Variable	SDF-1		
	AA/AG	GG	*p*
Number	121	131	
Adverse outcome (n, %)	16 (13.2)	9 (6.9)	0.092
Gender (male, %)	57 (47.1)	69 (52.7)	0.377
Age at transplant (year)	53.0 ± 10.5	51.8 ± 12.3	0.559
HLA mismatch (n, %)			
≤3	71 (65.7)	73 (61.3)	0.492
>3	37 (34.3)	46 (38.7)	
Parental DM (n, %)	10 (8.3)	13 (9.9)	0.648
Cigarette smoke (n, %)	31 (26.1)	39 (30.5)	0.441
HBV (n, %)	13 (10.7)	11 (8.4)	0.526
HCV (n, %)	14 (11.6)	7 (5.3)	0.074
Hypertension (n, %)	98 (81)	106 (80.9)	0.988
Cardiovascular event (n , %)	6 (5)	7 (5.3)	0.890
CVA	3 (2.5)	2 (1.5)	0.588
CAD	2 (1.7)	2 (1.5)	0.936
PAOD	2 (1.7)	4 (3.1)	0.466
Blood chemistry at Tx			
Glucose (g/dL)	95.6 ± 11.7	97.3 ± 12.4	0.494
Triglyceride (mg/dL)	123.6 ± 52.2	142.9 ± 77.7	0.187
Total cholesterol (mg/dL)	164.6 ± 35.9	163.6 ± 35.6	0.869
Albumin (g/dL)	4.2 ± 0.56	4.2 ± 0.48	0.862
Calcium (mg/dL)	9.0 ± 1.03	9.0 ± 1.05	0.890
Phosphate (mg/dL)	2.49 ± 1.39	2.54 ± 1.35	0.839
Body mass index (kg/m^2^)	22.0 ± 3.3	22.6 ± 3.6	0.183
Treatment (n, %)			
Tacrolimus	92 (76)	100 (76.3)	0.626
Cyclosporine	28 (23.1)	28 (21.4)	
Serum Creatinine post-Tx			
1 month	1.47 ± 1.02	1.45 ± 0.79	0.788
3 month	1.32 ± 0.38	0.33 ± 0.38	0.720
6 month	1.29 ± 0.43	1.32 ± 0.40	0.232
12 month	1.26 ± 0.48	1.28 ± 0.50	0.988
Δ12-6	−0.04 ± 0.36	−0.04 ± 0.34	0.707

Abbreviation: HLA, human leukocyte antigen; DM, diabetes mellitus; CVA, cerobrovascular accident; CAD, cardiovascular disease; PAOD, peripheral arterial occlusive disease.

**Table 2:** The risks of experiencing an adverse outcome with variant SDF-1 genotype and allele.

Variant	aHR	95% CI	*p*
GG	1		
AA/AG	2.742	1.106–6.799	0.03
G	1		
A	2.306	1.254–4.24	0.008

Abbreviation: aHR, adjusted hazard ratio; CI, confidence interval; Adjusted hazard ration was calculated by stepwise Cox regression analysis adjusted with age, gender, HLA, CV event, HCV infection, and usage of immunosuppression.

## 3. Discussion

In the current study, we report that non-DM RTRs who carried the SDF-1 genotype AA/AG or an allele A had a higher risk of an adverse long-term allograft outcome in comparison to those who had the SDF-1 GG genotype or a G allele.

SDF-1 is a highly potent chemo-attractant for CXCR4 positive monocytes and naïve T cells that is constitutively expressed by many cell types, including endothelial and dendritic cells. In addition to its role in T-lymphocyte migration, SDF-1 is also a co-stimulator of CD4^+^ T cells and plays a role in HIV progression [10,11], microvascular complications among systemic sclerosis patients [21], and progression of carotid artery stenosis [22].

Chemokines, which are known to be involved in leukocyte migration during tissue inflammation, are required for development and tissue homeostasis [8]. Stromal derived factor 1 (SDF-1), which is a 93 amino acid chemokine, was first described as a growth factor for pre-B cells but turned out to be an essential regulator for important functions, such as recruiting progenitor cells to hypoxic tissues, epithelial progenitor cell migration, angiogenesis, and natural killer cell/B cell developments. When SDF-1-CXCR4-related maladaptive control occurred, podocyte progenitors of the Bowman’s capsule would proliferate in crescentic glomerulonephritis [23], hypoxia-induced upregulation of CXCR4 in nephrosclerosis was observed [24], and there was insufficient podocyte regeneration in diabetic nephropathy [25]. Together, these results indicate that SDF-1-CXCR4 function is necessary to maintain homeostatic tissue renewal and regeneration upon renal injury [8].

Moreover, in an ischaemia/reperfusion injury mice model, Stokman *et al.* found that renal SDF-1 protein levels increased significantly in the early phase of kidney injury and a reduction of corticomedullary SDF-1 expression occurred, which was accompanied by severely increased tubular injury and decreased renal function after antisense treatment. They postulated a possible role of SDF-1 in mediating tubular epithelial cells against ischemic injury [26]. For chronic kidney disease, Chen *et al.* found that: SDF-1 mRNA doubled in human focal segmental glomerulosclerosis, which might be due to the use of angiotentin converting enzyme inhibitor; and local SDF-1 delivery induces glomerular eNOS activation in a CXCR4-dependent manner. They postulated that local SDF-1/CXCR4 could function to prevent renal fibrosis [27].

In addition to its association with infections, the inflammatory process, or acute and chronic kidney diseases, the SDF-1 3' A genotype of liver transplant recipients was found to contribute to a 36-month reduction in survival time. It is postulated that SDF-1 might promote integrin-mediated retention and adhesion of alloreactive lymphocytes to the biliary epithelium [14]. In fact, the portal tract appears to be the main target of infiltrating cells during acute rejection, meanwhile portal inflammation is associated with a progressive loss of bile ducts in the course of chronic rejection [14].

In studying human renal transplantation, Hoffmann *et al.* reported that recipients diagnosed with a chronic allograft rejection demonstrated an upregulation of SDF-1 in interstitial infiltrates and infiltrating neo-intimal cells of arteries [28]. Moreover, Gao *et al.* found that the anti-SDF-1 antibody could down-regulate the expression of SDF-1 and CXCR-4, and also delayed the progression of chronic allograft nephropathy in rats [17]. In this study, we found that SDF-1 AA/AG genotypes and the A allele of RTRs predisposed patients to impaired long-term allograft outcomes compared with those patients with GG genotype and G allele.

On the contrary, the CD26/SDF-1 axis is reported to be involved in the endothelial repair process following kidney transplantation [29]. Thus, future trials should evaluate whether SDF-1 has pro-inflammatory or protective properties in renal transplantation, as well as the possible underlying pathophysiology.

Since this was a retrospective single-center cohort study, one limitation of the current investigation was the number of patients. Additionally, this study lacked a functional assay evaluating SDF-1. Thus, further studies that include more patients and evaluate serum activities of SDF-1 are warranted.

## 4. Materials and Methods

### 4.1. Subjects

Between March 2000 and January 2008, RTRs who were diagnosed as pre-DM, PTDM and those who did not offer informed consents were excluded, resulting in the recruitment of a total of 252 RTRs. Pre-DM and PTDM RTRs were excluded since both conditions are known to significantly impair allograft outcome [20]. Clinical data including age, gender, human leukocyte antigen, HBV, HCV, parental DM, follow-up duration after transplantation, HTN, cardiovascular (CV) events (including stroke, coronary artery disease and peripheral artery occlusive disease) and usage of tacrolimus-based or cyclosporine-based immunosuppressive regimens were recorded. After transplantation, serum creatinine (SCr) was monitored at 1, 3, 6, and 12 months during regular outpatient follow up. The difference in SCr between 6 and 12 months after transplantation was defined as delta SCr.

The follow-up period began on the date of kidney transplantation and continued until a primary outcome or the end of the study (December 2009). The blood samples for determination of the SDF-1 genotype were obtained once informed consent was acquired from each RTR during regular follow up. This study was approved by the institutional review board of Chung Shan Medical University Hospital.

### 4.2. Genomic DNA Extraction

Genomic DNA was extracted from whole blood samples, which were collected from study subjects using QIAamp DNA blood mini kits (Qiagen, Valencia, CA, USA) in accordance with the manufacturer’s instructions. DNA was dissolved in TE buffer (10 mM Tris, pH 7.8; 1 mM EDTA) and then quantitated via measurement of optical density (OD260). The final preparation was stored at −20 °C and was used to create templates in the polymerase chain reaction.

### 4.3. Polymerase Chain Reaction—Restriction Fragment Length Polymorphism (PCR-RFLP)

The SDF-1 (rs1801157) gene polymorphism was determined by a PCR-RFLP assay. The sequences of primers used to amplify the SDF-1 genotype were 5'-CAGTCAACCTGGGCAAAGCC-3' and 5'-CCTGAGAGTCCTTTTGCGGG-3'. PCR was performed in a 10 μL reaction mixture containing 100 ng DNA template, 1.0 μL of 10× PCR buffer (Invitrogen, Carlsbad, CA, USA), 0.25 U of Taq DNA polymerase (Invitrogen), 0.2 mM deoxyribonucleotide triphosphates (dNTPs; Promega, Madison, WI, USA), and 200 nM of each primer (MDBio Inc., Taipei, Taiwan). The PCR cycling started at 94 °C for 5 min followed by 35 cycles of 94 °C for 1 min, 60 °C for 1 min, and 72 °C for 2 min, with a final step at 72 °C for 20 min to allow for a complete extension of all PCR fragments. The PCR products of SDF-1 gene polymorphisms were subjected to enzymatic digestion by incubation with *Hpa*II for 4 h at 37 °C and subsequent electrophoresis in 3% agarose gels.

### 4.4. Adverse Composite Outcome

The primary outcome in this study was the time to the first event including any of the following: doubling of SCr, graft failure, or death. Graft survival was calculated from the date of transplantation until the date of graft loss. Living or deceased patients with a functioning graft at date of last follow-up were right censored. Doubling of SCr, graft failure, and patient survival were chosen as the adverse composite outcome because they encompassed well-defined, clinically relevant outcomes to both patients and physicians [6,20].

### 4.5. Statistical Analysis

Hardy-Weinberg equilibrium (HWE) was assessed using a goodness-of-fit chi-square test for SDF-1. Distributions of alleles were calculated by direct counting. Results were presented as mean ± standard deviation or percentages, and were analyzed by Mann-Whitney U test or chi-square test, as appropriate. The cumulative incidence and difference between time-to-event probabilities of the adverse outcome was analyzed by Kaplan-Meier curves and log-rank test. Stepwise multivariate Cox regression analysis was used to calculate the hazard ratio of experiencing an adverse composite outcome according to the SDF-1 genetic polymorphism. Age, gender, HCV infection, CV events, and usage of immunosuppressive regimens were included as covariates in this analysis. A *p* value of less than 0.05 was considered to be statistically significant. Statistical analysis was performed using MedCalc Statistical Software version 12.7.2 (MedCalc Software bvba, Ostend, Belgium).

## 5. Conclusions

To our knowledge, our results revealed a unique immune-modulating role of SDF-1 genetic polymorphisms on the outcome of renal allograft, suggesting that SDF-1 AA/AG genotype and an A allele possessed an unfavorable impact on the long-term allograft outcome.
